# Recent Advances and Achievements in Nanomaterial-Based, and Structure Switchable Aptasensing Platforms for Ochratoxin A Detection

**DOI:** 10.3390/s131115187

**Published:** 2013-11-06

**Authors:** Akhtar Hayat, Cheng Yang, Amina Rhouati, Jean Louis Marty

**Affiliations:** 1 BIOMEM, Université de Perpignan, 52 Avenue Paul Alduy, Perpignan Cedex 66860, France; E-Mails: akhtarloona@gmail.com (A.H.); chemyang1979@hotmail.com (C.Y.); amina.rhouati@gmail.com (A.R.); 2 Department of Chemistry and Biomolecular Science, Clarkson University, Potsdam, NY 13699-5810, USA

**Keywords:** aptasensor, nanomaterials, aptamer-conjugated nanoparticles, structure switchable sensing, ochratoxin A

## Abstract

Aptamer-based bioreceptors that can easily adopt their surroundings have captured the attention of scientists from a wide spectrum of domains in designing highly sensitive, selective and structure switchable sensing assays. Through elaborate design and chemical functionalization, numerous aptamer-based assays have been developed that can switch their conformation upon incubation with target analyte, resulting in an enhanced output signal. To further lower the detection limits to picomolar levels, nanomaterials have attracted great interest in the design of aptamer-based sensing platforms. Associated to their unique properties, nanomaterials offer great promise for numerous aptasensing applications. This review will discuss current research activities in the aptasensing with typical example of detection of ochratoxin A (OTA). OTA, a secondary fungal metabolite, contaminates a variety of food commodities, and has several toxicological effects such as nephrotoxic, hepatotoxic, neurotoxic, teratogenic and immunotoxic activities. The review will introduce advances made in the methods of integrating nanomaterials in aptasensing, and will discuss current conformational switchable design strategies in aptasensor fabrication methodologies.

## 1. Introduction

Due to their enhanced specificity, selectivity and versatility biomolecular recognition phenomena have inspired many scientists to develop sensors based on these effects. As a consequence, numerous biosensors based on bio-recognition elements have been developed for various applications. However, a significant problem in the fabrication of these devices is that most of the biomolecules do not generate an easily measurable signal upon binding to the target analytes. For example, most commonly used bioreceptors such as enzymes and antibodies do not change their conformation or produce any specific signal upon incubation to their antigen. A comparative study of the properties of three commonly used biorecepters is provided in Table 1.

**Figure 1.**
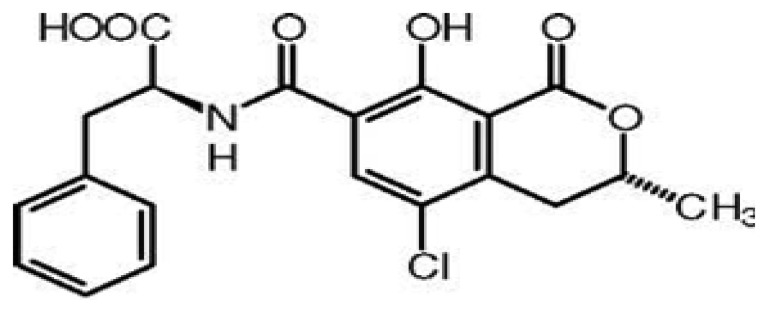
Chemical structure of ochratoxin A.

As a result, existing bio-analytical methods require many steps in the fabrication of sensors to get a measurable signal. Due to this, these methodologies are not well suited for use outside the laboratory, and hard to apply for real time and *in situ* applications. To circumvent these problems, sensors measuring a change in mass, charge or optical properties upon target binding to bioreceptors have been designed. However, they also suffer from non-specific adsorption, poor selectivity and interferences from the matrix [1,2]. Thanks to their Nature-learned process, aptamers have solved the problem of real-time sensing in complex environments. Aptamers, single strand oligonucleotides, have the potential to assist in the development of improved sensing technologies [3–5]. The aptamer-based assays rely on antigen binding-induced conformational changes or oligomerization states rather than binding assisted changes in adsorbed mass or charge. These switchable events lead to measurable signals, and inspired by this phenomena, significant interest has been shown in the fabrication of aptamer assays based on this principle [6]. However, a biosensing device requires two components, a biorecognition element and a signal transducer element [7]. On balance, the rapid development of nanoscale science and technology with the successful synthesis and characterization of a variety of nanomaterials has provided transducer surfaces with unique optical, electronic, magnetic and catalytic properties [8–13]. Nanomaterials are structures having a size range of 1 to 100 nm and are characterized by the properties different from their larger scale counterparts [14–16]. Nanomaterials have attracted significant attention in energy harvesting [15] and information technology [17]. Meanwhile, recently, researches have synthesized nanomaterials that are very well integrated in the fabrication of biosensors [18]. Both due to their enhanced biocompatibility and biofunctionality, nanomaterials can be very easily conjugated to synthetic or natural ligands and biomolecules [19]. Nanomaterials, including metallic nanoparticles, semiconductor nanocrystals (quantum dots), carbon nanotubes, nanorods and nanoshells have found widespread interest and applications in the biosensing technology field. Nanomaterials serve as signal transducers, as well as signal amplifiers in sensing platforms [8]. Meanwhile, aptamers possess excellent recognition properties. Thus the integration of nanomaterials into aptamer-based assays provides a potentially promising design of aptasensing platforms. This novel combination has resulted in the design of stimuli-responsive nanomaterial assemblies, and various bioassay formats have been developed for a wide range of target analytes [20–26]. To demonstrate our discussion, we review recent efforts to develop such assays for ochratoxin A (OTA) detection. OTA (Figure 1) is a low molecular weight mycotoxin produced by certain strains of filamentous fungi of *Aspergillus and Penicillium* [27,28] and detected in several food matrices [29–31]. Because of its widespread occurrence on a large variety of agricultural commodities and the potential health risks, mainly toward humans, OTA has been classified as a possible human carcinogen (group 2B) by the International Agency for Research on Cancer [32]. Our lab [33] and a group from Canada [34] have applied SELEX process for the screening of DNA aptamers against OTA. The most commonly used aptamer sequence for OTA is 5′-GATCGGGTGTGGGTGGCGTAAAGGGAGC ATCGGACA-3′). In this article, we attempt to cover major advances in structure-switchable and nano- materials-based aptamer assays, using OTA as a particular example of. Firstly, the advantages of structure-switchable bioassays, and various types of nanomaterials integrated in biosensing are reviewed. Finally, to demonstrate our discussion, aptamer assays based on conformational changes and nanomaterial integration are discussed in detail with OTA as the specific example.

## 2. Advantages of Structure-Switchable Aptamer Assays

Unlike enzymes and antibodies, nucleic acids are considered as biomolecular switches, as they can be reversibly shifted between two or more stable states in the presence of a ligand. This conformational change can be investigated in aptasensing techniques to transduce the biorecognition event between the aptamer and its target into a measurable signal [35,36]. In addition to the easily generated and highly specific signal response, biomolecular switches offer several advantages in the realm of biosensors. Structure-switching sensors are versatile and can be used for continuous and real time molecular monitoring in complex environments whether *in vitro* or *in vivo* [37]. This flexibility is due to the rapid, reversible and reagentless structure-switching. Conformational changes are mainly based on the formation of many weak and non-covalent bonds, such as hydrogen bonding, hydrophobic effects and van der Waals forces, resulting in a very high specificity [38]. Indeed, the optimization procedures are rapid, simple and they do not influence binding specificity, since the switching equilibrium is related to the switch's underlying thermodynamics. This equilibrium is also dependent on target concentration which allows a quantitative detection. Finally, switch-based aptamer assays can be adapted to optical, electrochemical or biochemical techniques for the detection of a wide range of analytes in different environments [2].

Upon binding to their targets, aptamers fold into a stable three dimensional structure. To undergo this binding-induced conformational change, aptamers are either destabilized or hybridized to a complementary sequence. The binding to the target leads in the first case to a change in the folding equilibrium constant, while a transition between double-stranded and aptamer fold will occur in the second case. Structure-switching aptasensing can be then achieved by conformation-linked changes in fluorescence emission, electron transfer or biochemical activity [39,40]. Conformation-linked fluorescence quenching is the most widely used method; it is based on a distance-dependent change either on the Forster Resonance Energy Transfer (FRET) between two fluorescent reporters, or on excimer formation. This can be also achieved via conformation-linked changes in the chemical environment around a single, structure-sensitive fluorophore [41]. Electroactive reporters such as ferrocene are used in case of switch-based electrochemical sensors. The labeled biomolecule is immobilized onto an electrode surface and a measurable electrochemical signal is obtained after the binding-induced conformational changes which alter the redox current produced by the reporter [42]. Finally, a biochemical signal can also be produced by structure-switching via activation or inhibition of a second reporter (e.g., enzyme) [43].

## 3. Nanomaterials for Aptasensing

Because of the quantum size effects stemming from the high surface area to volume ratio, nanomaterials possess unique optical, electronic, magnetic, mechanical, physical and chemical properties. Recently, investigators have prepared nanomaterials of extensive interest for bioanalysis and bioassays. Specifically increasing applications have been found in biosensor development. Among the different biosensors, the ones using aptamers as biorecognition elements have been coined as “aptasensors”. Nowadays, numerous nanomaterials had been successfully synthesized for aptasensing applications due to rapid developments in nanotechnology [44–46]. Herein, we emphasize the most commonly used nanomaterials such as gold nanoparticles, carbon-based nanomaterials, quantum dots and magnetic nanoparticles for aptasensing applications.

### 3.1. Gold Nanoparticles

Gold nanoparticles (AuNPs) as a colorimetric indicator have become very attractive in DNA-related colorimetric assays due to their high extinction coefficients and distance-dependent optical properties. AuNPs exhibit surface plasmon resonance (SPR) at specific incident light wavelengths due to the collective oscillation of free electrons in their conduction bands, exhibiting intense surface plasmon absorption bands [47]. In addition, the SPR band of gold nanoparticles has strong distance-dependent properties. Colorimetric sensors using AuNPs have been widely explored and have important applications [48]. However, the modification of DNA onto AuNPs and the separation of the modified AuNPs from excess mercaptoalkyl-oligonucleotides were often required. Hence, investigators developed unmodified AuNPs-based colorimetric biosensors to simplify the detection process. A single stranded DNA (aptamer) uncoils sufficiently to expose its bases, which easily adsorb onto the surface of AuNPs. Thus, a DNA phosphate backbone with a large number of negative charges exists on the outside of AuNPs. The electrostatic repulsion prevents the strong van der Waals attraction and enhances the stability of AuNPs against salt-induced aggregation. In the presence of target molecules, the aptamer binds with the target and forms a rigid structure, which prevents the exposure of the aptamer bases to AuNPs, and thus loses the ability to protect the AuNPs under high-salt conditions [49–51].

### 3.2. Carbon-Based Nanomaterials

Carbon-based nanomaterials (CBNs) include single or multi-walled nanotubes, fullerenes, nanodiamonds, and graphene. Recent research has found that carbon nanotubes can act cooperatively as effective quenchers for a variety of fluorophores through an energy or electron transfer process, resulting in low background and high signal-to-noise ratio for their use in biosensor development. Due to the π-π stacking interaction between the DNA bases of the aptamer and the carbon nanotubes, the fluorescent dye and the multi-walled nanotubes are brought into close proximity, which leads to fluorescence quenching. However, with further addition of target molecule, specific binding of the aptamer to target drives the aptamer away from the surface of the multiwalled nanotubes and restores the quenched fluorescence. This recovered fluorescence intensity is found to be in linear proportion to the concentration of target [52–54].

### 3.3. Quantum Dots

Fluorescent semiconductor quantum dots (QDs)—aptamer conjugates have been emerged as a powerful new sensing platform showing great potential in biosensing. Quantum dots are semiconductors whose electronic characteristics are closely related to the size and shape of the individual crystal. The QDs-aptamer conjugate sensors are based on the emission changes of QDs due to fluorescence resonance energy transfer (FRET) or photoinduced electron transfer. QDs are often used as FRET donors and their emission can be size-tuned to optimize spectral overlap with acceptor dyes. Different-colored QDs can be excited by a single light source, yet produce specific, narrow and symmetric emissions of different colors, which is very useful for multiplexing [55,56].

### 3.4. Magnetic Nanoparticles

A biosensing magnetic nanoparticles (MNPs) platform is comprised of an inorganic nanoparticle core and a biocompatible surface coating. The composition of these inorganic nanocrystals ranges from metals and alloys to metal oxides. During the past decade, superparamagnetic iron oxide nanoparticles have been a major research focus [57–59]. A biocompatible surface coating not only has a specific biological selectivity, but also stabilizes the iron oxide particles and prevents aggregation in both a biological medium and a magnetic field. Biological functions of MNPs with aptamers enable selective target extraction or their use as contrast agents for magnetic resonance imaging (MRI) [60–62].

## 4. Nano Materials Integrated, and Structure Switchable Aptamer Based Assays for OTA Analysis

As mycotoxin contamination occurs at trace levels, highly sensitive and accurate methods are required for OTA detection. Aptamer-based methods have emerged as a powerful alternative to classical and existing biochemical methods in recent years [63,64]. Aptamers are short single stranded oligonucleotides engineered through a process known as SELEX. In this screening process, a random sequence oligonucleotide library is incubated with target analyte of interest and sequences with strong affinity to the target are separated from the unbound species using a suitable partitioning method. The sequence of the selected candidate with higher affinity to the target analyte is amplified through PCR. After the identification of an appropriate sequence, an aptamer is synthesized by a chemical process [65]. The various aptamer assays based on nanomaterials and conformational changes of aptamers for OTA analysis developed during these last years are described below and summarized in Tables 2 and 3, respectively.

### 4.1. Nanomaterials Integrated Aptamer Based Assays for OTA

Whereas a large number of nanomaterials-based assays have been developed for OTA analysis, we have categorized these strategies based on the specific function of nanomaterials in a given assay.

#### 4.1.1. Functionalized Nanoparticles as Immobilization Support

Due their large surface area and unique magnetic properties, magnetic nanoparticles hold great potential to be used as immobilization support in biosensing. The first OTA aptasensor based on magnetic nanoparticles as immobilization support was developed by Bonel *et al.* in 2011 [72]. Paramagnetic microparticle beads (MBs) were functionalized with the aptamer, and were allowed to compete with a solution of the mycotoxin conjugated to the enzyme horseradish peroxidase (OTA-HRP) and free OTA. After separation and washing steps, the modified MBs were placed on a screen printed carbon electrode used as transduction surface under a magnetic field. Differential pulse voltammetry was used to detect the enzymatic reaction with the substrate. Moreover, the authors also tested gold nanoparticles-modified aptamers for preliminary experiments. The designed sensing platform with magnetic nanoparticles provided a linear response to OTA in the range of 0.78–8.74 μg/L, and a limit of detection of 0.07 μg/L was found. The method was validated with certified and spiked wheat samples. However, the use of HRP as label is subject to non-specific adsorption on the transducer surface which may result in an elevated non-specific signal. Alternatively, our group [74] designed an electrochemical aptasensor based on magnetic attachment of magnetic nanoparticles on disposable screen-printed electrodes for the detection of OTA. Functionalized super- paramagnetic nanoparticles were used to perform indirect and direct competitive assays. Alkaline phosphatase enzyme was used as label to get the electrochemical signals. The performance of the optimized aptasensors in terms of reproducibility, stability, sensitivity, and analysis time was studied. The detection was performed by employing differential pulse voltammetry. The aptasensor obtained using this novel approach allowed us to detect OTA at a level of 0.11 μg/L, with a linear range of 0.11–15 μg/L. The method was also validated for real wine sample analysis. Although both the devices based on magnetic nanoparticles were successfully employed to detect low levels of OTA in real samples, the incorporation of magnetic nanoparticles in a flow system was expected to offer attractive advantages over a batch system. Based on this idea, our group [78] constructed a fully automated flow electrochemical aptasensor based on the integration of magnetic nanoparticles and selective capture of aptamers in a flow system. The developed flow-based aptasensor was based on continuous/stopped flow conditions for the detection of ochratoxin A (OTA) employing direct and indirect competitive strategies. A detection limit of (0.05 μg/L) was obtained with the indirect flow-based aptasensor. Finally, the flow-based aptasensor was validated with real beer samples, indicating good recovery values. As expected, the integration of magnetic nanoparticles as immobilization support in a flow system enhanced the sensitivity of the method. Recently, Zhang *et al.* [80] reported a signal on fluorescent aptasensor based on Tb^3+^, structure-switching aptamer for OTA analysis. Streptavidin-modified magnetic nanoparticles were used to immobilize the specific sequence of biotin-labeled anti-OTA aptamer. Incubation of two single-stranded signal probes resulted in hybridization with anti-OTA aptamer to form a duplex structure. Knowing that single-stranded oligonucleotides enhance the emission of Tb^3+^ in solution but duplexes do not, the OTA binding with aptamer to form OTA-aptamer G-quadruplex released two single-stranded signal probes. Through magnetic separation, the released single-stranded signal probes left in the supernatant liquid increased the fluorescent intensity of Tb^3+^. Based on above concept, this aptasensor was able to detect as low as 0.02 μg/L OTA, with a linear range of 0.1–1 μg/L. The developed assay was used to detect OTA in wheat samples.

#### 4.1.2. Nanoparticles as Signal Generating Probe

Many nanomaterials are considered as ideal signal generating probes due to their intrinsic physicochemical properties [91]. Wang *et al.* [68] were the first ones to report a fluorescent strip sensor employing an aptamer-quantum dots technology to detect OTA. The limit of detection (LOD) for the fluorescent strip was 1.9 μg/L, and linear range was 0 to 10 μg/L, with a very short analysis time. The designed strategy was tested with real red wine samples. Later on, our group [69] introduced a novel aptamer-based technique to detect OTA using for the first time gold nanoparticles (AuNPs) as colorimetric indicators. In the presence of OTA, the conformation of OTA's aptamer in phosphate buffer saline changed from a random coil structure to a compact rigid antiparallel G-quadruplex structure. The quadruples structure resulted in salt-induced aggregation of AuNPs, and thus the color was changed from red to blue. The developed assays showed a linear range of 8.07–252.38 μg/L and detection limit of 8.07 μg/L was calculated. The same year, Wang *et al.* [71] integrated AuNPs in an aptamer-based chromatographic strip assay method for rapid OTA toxin detection. The aptamer-based assay was based on the competitive reaction between the DNA probe 1 and the target OTA to combine with aptamers. In the presence of OTA, it combined with aptamer-GNP probe, decreasing the aptamer-GNP and the red color intensity. The authors assigned a visual limit of detection 1 μg/L of the strip for qualitative detection, while the LOD for semi-quantitative detection could go down to 0.18 μg/L by using a scanning reader. Additionally, the method offered other advantages such as short analysis times and reduced interference effects contributing to good recovery values. To demonstrate the practical applicability and accuracy of the assay, the method was applied to analyze OTA in red wine samples. Similarly, Wu *et al.* [73] fabricated an aptamer assay using upconversion nanoparticles (UCNPs) as luminescent markers. The assay was based on aptamer-conjugated magnetic nanoparticles (MNPs) as the recognition and concentration element and upconversion nanoparticles (UCNPs) as highly sensitive labels. The bioassay system was designed by immobilizing DNA1 onto the surface of Fe_3_O_4_ MNPs to capture and concentrate OTA from bulk samples. In the presence of OTA, DNA1 released from the UCNPs modified DNA2 and resulted in a decrease in the luminescent signal. The decreased luminescent intensity was proportional to the concentration of OTA in the range of 0.0001–1 μg/L with a detection limit of 0.0001 μg/L. The proposed method then was successfully applied to measure OTA in naturally contaminated maize samples. Moreover in 2012, Tong *et al.* [76] combined magnetic nanoparticles, quantum dots and rolling circle amplification (RCA) to develop an electrochemical aptamer method for the detection of OTA. An amino-modified capture DNA was immobilized onto magnetic particles to hybridize with aptamer and a phosphate labeled padlock DNA. The aptamer was dissociated from the bioconjugate in the presence of OTA, and the padlock DNA subsequently was hybridized with the capture DNA to form a circular template with the aid of the T4 ligase. Next, capture DNA generated a long tandem repeated sequences by phi29 DNA polymerase and dNTPs. Finally, two quantum dot (QD)-labeled DNA probes were tagged on the resulting RCA product to indicate the OTA recognition event by electrochemical readout. This strategy detected OTA down to a level of 0.0002 μg/L with a linear range of 0.0005–10 μg/L. The proposed approach was tested by determining OTA in red wines. Recently, Hun *et al.* [79] used functionalized carboxysilica nanoparticles as chemiluminescent labels in the design of OTA aptamer-based assays. In the given sensing platform, the strands of OTA aptamer (DNA1) and OTA aptamer complementary (DNA2) were immobilized onto magnetic particles. The presence of OTA resulted in dissociation of DNA2, with release of MB. The released DNA2 then hybridized with DNA3 and the formation of double stranded DNA was cleaved by nicking endonuclease Nb.BbvCI, and a short single-stranded DNA was produced. The cleaved DNA strand generated a new site by Phi 29 DNA polymerase. Finally, using DNA and a carboxysilica nanoparticles chemiluminescence (CL) probe, the short single-stranded DNA was detected. The method exhibited a linear range of 0.001–15 μg/L, with a detection limit of 0.0003 μg/L. The developed method has been used to measure OTA in naturally contaminated wheat samples.

#### 4.1.3. Nanomaterials as Fluorescent Quencher

**Figure 2.**
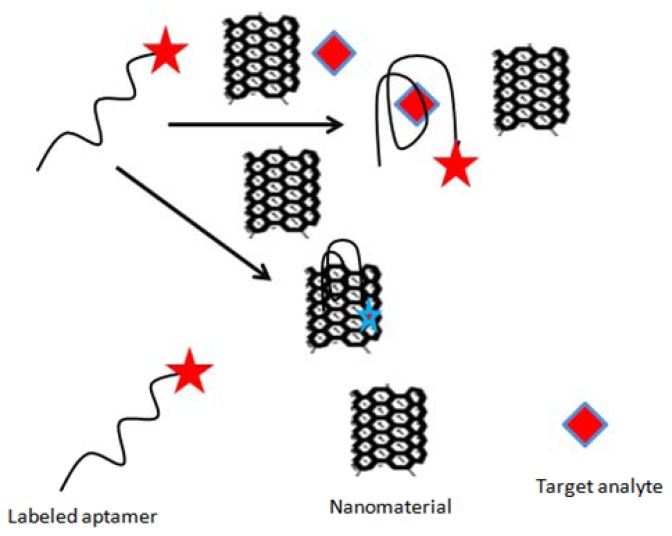
Single-walled carbon nanotubes based quenching of free FAM-aptamer for selective determination of ochratoxin A [75].

In addition to playing a role of signal generator probe, some of the nanomaterials can also function as fluorescent quenchers. Based on this idea, Guo *et al.* [75] constructed a sensitive and selective fluorescent aptasensor for ochratoxin A (OTA) detection. The authors used single-walled carbon nanotubes (SWNTs) as quencher, which were able to quench the fluorescence of free unfolded toxin-specific aptamer attached with FAM (carboxyfluorescein, Figure 2). The detection limit obtained with the assays was 0.007 μg/L, with a linear range from 0.02–3.0 μg/L. The method was assessed for possible interferences, and applied to detect OTA in 1% beer samples containing buffer solution spiked with a series of concentrations of OTA. Similarly Duan *et al.* [77] designed a sensitive and specific fluorescence energy transfer aptasensor based on dye-tagged ssDNA hybridized with aptamer-conjugated gold nanoparticles. The binding between the aptamer-Au NPs conjugate and the dye-labeled ssDNA resulted in the fluorescence quenching of FAM due to its close proximity. The addition of OTA led to an increase in fluorescence signal, attributed to the formation of a quadruplex-OTA complex, which detached from the surface of AuNPs. The method showed a detection limit of 0.002 μg/L, with a linear range of 0.005 to 5 μg/L. The proposed method was successfully applied to measure the concentration of OTA in naturally contaminated maize samples.

#### 4.1.4. Nanomaterials for Signal Amplification

Since OTA is present at trace levels in real samples, high sensitivity is required for aptamer assays in practical applications. To improve the sensitivity of the assays, an amplification system is required. Nanomaterials have attracted great interest in bioassays to amplify the generated signal. Kuang *et al.* 2010 [66] developed an ultrasensitive and rapid electrochemical platform for the specific detection of OTA using gold nanoparticles as signal amplifiers. In the proposed strategy, binding of the OTA target analyte to the aptamer changed the redox current of methylene blue (MB), which was used as the electrochemical probe, in a manner that was dependent on OTA concentration. A gold nanoparticles- functionalized DNA strand was used to amplify the electrochemical signal. The method showed a detection limit as low as 0.03 μg/L, and the linear range was from 0.1 to 20 μg/L. To demonstrate the applicability, the assay was tested with red grape wine samples. Later on in the same year, Wang *et al.* [67] designed a selective electrochemiluminescent biosensor integrating gold nanoparticles to enhance the signal. The platform was constructed by using a DNA aptamer as the recognition element and *N*-(4-aminobutyl)-*N*-ethylisoluminol as the signal-producing compound. The electrochemiluminescent aptamer biosensor was fabricated by immobilizing aptamer complementary DNA 1 sequence onto the surface of a gold-nanoparticle (AuNP)-modified gold electrode. A decrease in electrochemiluminescence (ECL) signal upon aptamer-analyte binding was used to monitor the OTA concentration. The decreased ECL intensity was proportional to an OTA concentration ranging from 0.02 to 3.0 μg/L, with a detection limit of 0.007 μg/L. The proposed method has been applied to measure OTA in naturally contaminated wheat samples. Similarly, Evtugyn *et al.* [81] designed an electrochemical aptasensor for ochratoxin A (OTA) detection by using a gold electrode covered with electropolymerized neutral red and silver nanoparticles obtained by chemical reduction with macrocyclic ligands bearing catechol fragments. Herein, silver nanoparticles were used to amplify the electrochemical signal. The interaction of OTA induced the conformational switch of aptamer, which increased the charge transfer resistance. The obtained LOD was 0.02 μg/L, with a linear range of 0.048–0.807 μg/L. The aptasensor was validated with spiked beer samples.

#### 4.1.5. Aptamer-Modified Nanomaterials as Solid Phase Extraction Sorbent for OTA Analysis

Knowing the strong surface attachment of biomolecules on nanomaterials, Wu *et al.* [70] proposed the development of an oligosorbent for the selective extraction of OTA from complex samples with high efficiency. The platform was based on the aptamer immobilization on magnetite nanospheres surface. The proposed SPE method was robust, easy to prepare and cheaper than classic immunoaffinity cartridges. The technique was validated with different food samples and the obtained data was compared with C18 extraction.

## 5. Structure Switchable Aptamer Based Assays for OTA

Due to the diversity of detection methods employed, we categorize the structure switchable aptamer assays based on the types of output signal, *i.e.*, colorimetric, fluorescence and electrochemical signals.

### 5.1. Colorimetric Detection

**Figure 3.**
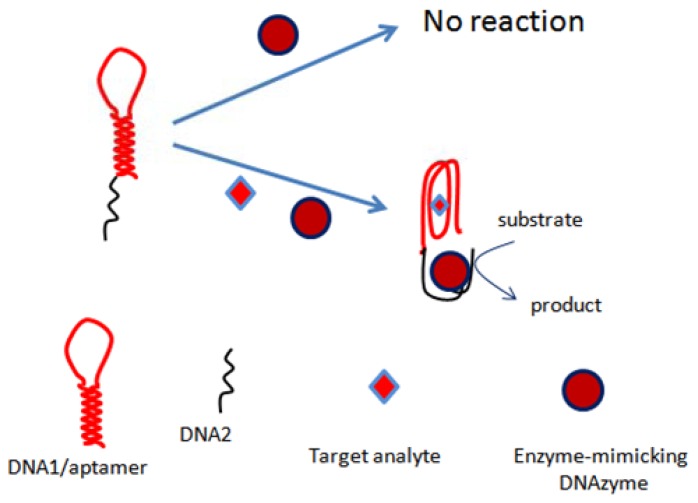
Aptamer-DNAzyme hairpins for biosensing of ochratoxin A [84].

The first colorimetric assay based on the conformational changes of OTA aptamer was reported by our group in 2012 [84]. The assay was based on a nucleic acid hairpin structure. The hairpin structure comprised a OTA-specific aptamer and a G-rich sequence of nucleotides mimicking HRP activity. It was shown in the assays that several species like Mg^2+^ and K^+^ have an effect on the activity of DNAzyme as well as on the OTA-aptamer recognition phenomena. The assay principle was based on the opening of hairpins in the presence of OTA due to the formation aptamer-analyte complex, which resulted in self-assembly of the active HRP-mimicking DNAzyme (Figure 3). The activity of this DNAzyme was linearly correlated with OTA concentration up to 4 μg/L, showing a limit of detection of 1 μg/L, and the method was demonstrated with wine samples. Similarly, another colorimetric bioassay exploring the conformational changes of aptamer was proposed by our group [88]. The assay relied on the self-assembly of DNAzyme-aptamer conjugates. The binding of OTA to aptamer led to a decrease in the hybridization efficiency, thus increasing the DNAzyme activity. The activity of DNAzyme was directly related to OTA concentration in a linear range up to 12.11 μg/L, with a detection limit of 1.61 μg/L. The proposed method was employed to detect OTA in wine samples.

### 5.2. Fluorescence Detection

Fluorescence has been widely applied in biosensing applications due to its various analytical advantages. By combining fluorescent labels with DNA, researchers have developed many fluorescence-based platforms. Sheng *et al.* [83] designed a platform in which FAM-modified aptamer was adsorbed onto the basal plane of graphene oxide via the π–π stacking force in the absence of OTA. Consequently, the fluorescence of FAM was quenched readily via energy transfer from dye to graphene oxide. However, the presence of target analyte (OTA) induces conformational changes of the aptamer, leading to the formation of an antiparallel G-quadruplex which was resistant to the adsorption onto the larger planar surface of graphene oxide. Based on this principle, the fluorescent intensity as a function of ochratoxin A concentration was correspondingly measured. The method showed a detection limit of 8.8 μg/L, and linear range of 807–14,133 μg/L, and was tested with red wine samples. Later on, Duan *et al.* [82] established a new switchable fluorescence method for the detection of OTA. In the designed strategy, the binding of immobilized aptamer to the target OTA induced the conformation change of the aptamer, and resulted in the dissociation of the FAM-tagged complementary DNA chain from the aptamer, finally leading to a fluorescent signal change. Based on the above experiments, the authors have detected OTA in the linear range of 0.002–10 μg/L, with a detection limit of 0.001 μg/L. The proposed method was validated with corn flour samples. Recently, Chen *et al.* [85] have designed a simple and rapid sensing platform for the highly sensitive and selective detection of OTA based on a target-induced structure-switching signaling aptamer. The method was based on the OTA-induced conformational change of DNA aptamer duplex to ligand-aptamer complex, which led to the release of hybridized quencher-tagged DNA strand from the fluorescein-labeled OTA aptamer, generating a substantially increased fluorescence intensity. The assay exhibited a linear range of 1 to 100 μg/L, with a detection limit of 0.8 μg/L. The method was also illustrated with interference studies, and was used for quantitative detection of OTA in corn samples.

### 5.3. Electrochemical Detection

**Figure 4.**
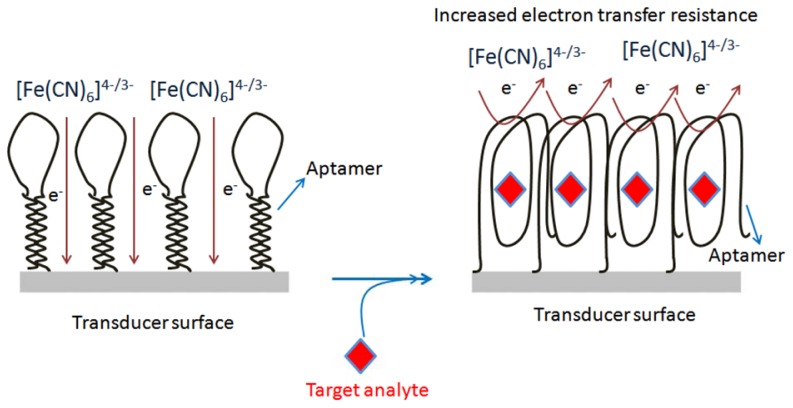
Design of PEG-aptamer two piece macromolecules integrated sensing platforms [89].

Aptamers have the ability to fold into a well-defined three dimensional structure upon binding to target analytes, which facilitates the probing of complex formation by the electron transfer features of the redox moieties. Based on this knowledge, Zhang *et al.* [86] reported an electrochemical DNA biosensor based on a hairpin anti-OTA aptamer and site-specific DNA cleavage of the restriction endonuclease TaqaI. TaqaI was used to specifically cleave only double strand DNA, but not single strand DNA. The hairpin-DNA aptamer probe (Hap), thiolated single strand DNA labeled with biotin groups, was immobilized on a gold electrode. The OTA incubation resulted in a Hap-OTA G-quadruplex, with opening of the stem of Hap. The resulting HRP-tagged Hap-OTA catalyzed the hydrogen peroxide (H_2_O_2_)-mediated oxidation of 3,3′,5,5′-tetramethylbenzidine sulfate (TMB) after reaction with the streptavidin-HRP, accompanied by a change of the solution color from colorless to blue, and an increased electrochemical current signal. By employing the above design, this DNA biosensor detected as low as 0.0004 μg/L of OTA, with a linear range of 0.001–0.02 μg/L. The method was applied to wheat samples. The same group [87] developed a one-step electrochemical aptasensor for rapid and ultrasensitive OTA detection by employing the principle of the binding-induced conformational change of the aptamer against OTA. The developed electrochemical aptasensor method permitted the detection of OTA with a sensitivity of 0.000095 μg/L as the LOD, and 0.0001 to 0.001 μg/L as linear range. The assay was used to monitor the OTA in red wine samples. Recently, our group has designed two strategies to construct structure-switchable electrochemical label-free aptasensors for small size molecule detection. The fabrication design for two aptasensors was demonstrated by the detection of OTA. In the first strategy, a long spacer chain of polyethylene glycol (PEG) was immobilized on a screen printed carbon electrode (SPCE) via electrochemical oxidation of its terminal amino-group, and subsequently the amino-aptamer was immobilized on the modified surface to form two piece macromolecules. The designed immobilized macromolecules resulted in the formation of long tunnels on the SPCE surface, while the aptamer acted as the gate of the tunnels. The aptamer gates were closed due to change in conformation of aptamer upon target analyte binding, decreasing the electrochemical signal (Figure 4). The decrease in electrochemical signal was linear in the range of 0.00012–0.0055 μg/L, with detection limit of 0.00012 μg/L. This method was tested with beer samples [89]. The second platform was based on the direct chemistry of hexamethylene-diamine *via* electrochemical oxidation of its terminal amino-group on SCPE. The activated carboxy-aptamer was covalently linked to another amino terminal group of immobilized hexamethyldiamine to design a structure-switchable aptasensor for OTA detection. The decrease in electrochemical signal due to aptamer-analyte binding was used to measure the OTA concentration. The aptasensor showed a limit of detection of 0.1 μg/L with linear range from 0.12 to 8.5 μg/L, and was validated with beer samples [90].

## 6. Conclusions and Outlook

Nanomaterials with intrinsic physicochemical properties provide a powerful tool to design a variety of aptasensors. Structure-switchable aptamer-based assays offer a rapid and selective way of transducing affinity binding events into an output signal. These assays are easy to perform, and do not require too many exogenous reagents. Owing to their nanoscale size and potential to work reversibly, structure-switchable aptamer-based assays are very well adopted for the continuous and real time monitoring of many target analytes in very complex environments. Despite these attractive advantages, aptamer-based assays are still in development phase as compared to immunoassays. The primary hurdles are the limited number of available aptamers and relatively poor knowledge of aptamer immobilization strategies. Moreover, despite the advantages over other bioreceptors, there is still a set of challenges that have hampered the commercialization of aptamers. A major drawback is the small molecule aptamer development and at the point of K*_d_* determination. Because of this problem associated with *K_d_* measurement, several complementary methods may be required to improve the binding affinity of aptamers with their targets. However, recent years have witnessed important and rapid advances in the sequences of new aptamers, along with integration of new nanomaterials in aptamer-based assays. Material scientists are looking to explore new functional nanomaterials by employing different synthetic procedures. Although large numbers of new nanomaterials have been developed, the current state of the art of aptamer-conjugated nanomaterials is limited by the difficulty in bioconjugation chemistry, and the lack of intrinsic properties and functional moieties in some of the nanomaterials. To overcome these challenges, researchers are rapidly improving the existing procedures, maximizing the advantages of nanomaterials. Considering the rapid progress in the preparation of versatile nanomaterials, we predict that integration of these novel materials in structure- switchable aptamers will drive numerous advances in bioanalytical systems for clinical, environmental, food and industrial applications.

Similarly, after the design of OTA aptamers, many aptasensors integrating nanomaterials have been reported for the detection of OTA. Aside from the some of nanomaterials (gold nanoparticles, magnetic nanoparticles), many other nanomaterials are not extensively explored or are yet to be explored in the development of aptasensors for OTA analysis. One of the future aspects could be fabrication of aptamer-based assays by employing such unexplored nanomaterials for the detection of OTA. Similarly, aptamers for other mycotoxins, including fumonisin B1 [92], aflatoxin B1 [93,94] and zeeralenone [95] have been reported in the literature recently. Another area of opportunity could be extension of OTA nanomaterial strategies to design aptamer-based assays for the abovementioned molecules.

## Figures and Tables

**Table 1.** Comparative study of the properties of the three commonly used biorecepters.

**Property**	**Enzyme**	**Antibody**	**Aptamer**
**Acquisition**	*In vitro* process no animals are involved	*In vivo* process -animals are involved	*In vitro* process no animals are involved
	All the toxins don't inhibit enzymatic activity or not substrate of enzyme	Difficult to obtain antibodies against targets that are non-immunogenic or toxic	Possible to obtain aptamers against targets that are non-immunogenic or toxic
	Manipulation is not possible Physiological conditions obligatory	Manipulation of selection hardly or not possible Physiological conditions obligatory	Manipulate selection to obtain binding and kinetic properties desirable for specific assays Non-physiological conditions acceptable
		Difficult to expose different epitopes of the same target for selection Identification laborious the immunogen must be the major fraction in the immunization reagent	Expose different epitopes of the same target for selection Identification easy and rapid process performed on automated platform the target used for selection can be a small portion in the target preparation

**Stability**	Increased by modifications	Cannot be increased by modification	Increased by modifications
	Loss activity over time Narrow stability in terms of pH, ionic strength and temperature	Relatively stable over time Narrow stability in terms of pH, ionic strength and temperature	Relatively stable over time Stability over a wide range of pH, ionic strength and temperature
	Unstable at room temperature	Unstable at room temperature	Long room temperature shelf lives

**Specificity and selectivity**	Less selective and specific Can be engineered	Binding constants for target species comparable with aptamers	Binding constants for target species comparable with antibodies

**Modifications**	Can be engineered	Biological and hardly engineered	Chemically easy to engineer Side specific attachment
			Efficient and exact modification - of reporter molecule - of spacers - of functional groups
			Homogeneous product

**Denaturation**	Enzyme accelerates the reaction and does not consume	Mild conditions needed to prevent irreversible denaturation	Regeneration after denaturation possible
		Sometimes difficult to separate from antibody-target-complex	Easy to separate from aptamer-target-complex

**Sensors**	Immobilization at defined densities and locations difficult	Immobilization at defined densities and locations sometimes difficult	Immobilization at defined densities at precise locations on solid surfaces (microarrays)
	Immobilization can denature enzyme	Molecular recognition functionalities hardly possible	Conformational changes on binding providing molecular-recognition functionalities Irreversible
		Irreversible cross-linking usually not possible	cross-linking with target protein possible-second legand for detection not needed

**Table 2.** Nanomaterials integrated aptamer-based assays for OTA.

**Sr. No**	**Type of Nanomaterials Used**	**Function of Nanomaterial**	**LOD (μg/L)**	**Linear Range (μg/L)**	**Sample Analyzed**	**Reference**
1	Gold nanoparticles	Electrochemical signal amplification	0.03	0.1–20	Red grape wine	[66]
2	Gold nanoparticles	Electrochemiluminescent signal amplification	0.007	0.02–3.0	Wheat	[67]
3	Quantum-dots	Fluorescent signal generation	1.9	0–10	Red wine	[68]
4	Gold nanoparticles	Colorimetric signal generation	8.07	8.07–252.38		[69]
5	Magnetic nanoshpere	Modified nanospheres as solid phase extraction sorbent			Variety of food samples	[70]
6	Gold nanoparticles	Colorimetric signal generation	0.18		Red Wine	[71]
7	Magnetic nanoparticles	Immobilization support	0.07	0.78–8.74	Wheat	[72]
8	Upconversion nanoparticles Magnetic nanoparticles	Upconversion nanoparticles as luminescent marker, Magnetic particles as immoblization support	0.0001	0.0001–1	Maize	[73]
9	Magnetic nanoparticles	Immobilization support	0.11	0.11–15	Wine	[74]
10	Single-walled carbon nanotubes	Fluorescent quencher	9.73	10.09–80.76	Beer	[75]
11	Quantum dots, Magnetic nanoparticles	Quantum dots as electrochemical label, Magnetic nanoparticles as immbolization support	0.0002	0.0005–10	Red wine	[76]
12	Gold nanoparticles	Fluorescent signal	0.002	0.005–5	Maize	[77]
13	Magnetic nanoparticles	Immobilization support in flow system	0.05		Beer	[78]
14	Carboxy silica nanoparticles, Magnetic nanoparticles	Functionalized carboxy silica nanoparticles as chemiluminescent label, Magnetic nanoparticles as immbolization support	0.0003	0.001–15	Wheat	[79]
15	Magnetic nanoparticles	Immobilization support	0.02	0.1–1	Wheat	[80]
16	Electropolymeriz ed neutral red and silver nanoparticles	Electrochemical signal amplification	0.02	0.048–0.807	Beer	[81]

**Table 3.** Structure switchable aptamer based assays for OTA.

**Sr. No**	**Detection Method**	**Limit of Detection**	**Linear Range**	**Sample Analyzed**	**Reference**
1	Fluorescence detection	0.001	0.002–10	Corn flour	[82]
2	Fluorescence detection	8.8	807–14133	Red wine	[83]
3	Colorimetric detection	1	1–4	Wine	[84]
4	Fluorescence detection	0.8	1–100	Corn	[85]
5	Electrochemical detection	0.0004	0.001–0.02	Wheat	[86]
6	Electrochemical detection	0.000095	0.0001–0.001	Red wine	[87]
7	Colorimetric detection	1.61	1.61–12.11	Wine	[88]
8	Electrochemical detection	0.00012	0.00012–0.0055	Beer	[89]
9	Electrochemical detection	0.1	0.12–8 .5	Beer	[90]
